# Cervical spine injury after virtual reality gaming: a case report

**DOI:** 10.1186/s13256-021-02880-9

**Published:** 2021-05-31

**Authors:** D. Baur, C. Pfeifle, C. E. Heyde

**Affiliations:** University hospital, 04103 Leipzig, Germany

**Keywords:** Spine, Gaming, Case report, Spinal trauma, Virtual reality

## Abstract

**Background:**

We report a patient who fractured the seventh cervical vertebra while playing a virtual reality (VR) game, without any other trauma.

This case report aims to describe the spinal trauma incurred during the use of a VR headset in a video game.

**Case presentation:**

The Caucasian patient presented with pain and swelling in the lower cervical spine at our clinic after playing a video game involving a combination of shoulder, arm and head movements while wearing a VR headset. Preexisting comorbidities were not present in the 31-year-old male. No history of regular medication use or drug abuse was recorded. After performing a clinical examination and radiological diagnostics, we found a dislocated traumatic fracture of the spinous process of the seventh cervical vertebra. After a soft tissue defect was excluded through magnetic resonance imaging (MRI) diagnostics, a conservative therapy regimen with pain therapy and immobilization was started.

After hospitalization, outpatient controls were conducted at 4, 6 and 12 weeks. At 6 weeks after hospitalization, the patient had recovered from the injury without complications.

**Conclusions:**

Rapid movements during VR gaming can lead to injuries of the cervical spine. In addition to rapid movements, the additional weight of the VR headset as well as the decoupling of audiovisual stimuli from the perceived proprioceptive information should be considered. Determining whether this is an isolated incident induced by unknown preexisting factors or whether the trauma mechanism alone can lead to severe spinal trauma needs to be studied further with additional cases.

## Background

To determine the mechanism of trauma, it is essential to assess the equipment used. Virtual reality (VR) headsets portray a virtual world through special lenses [1]. The weight of commonly used devices, similar to the one that the patient in this report used, ranges from 470 to 610 g [2]. Different applications require different extents of movement in the neck and head area, as well as the rest of the body, and the motions of extremities are often tracked simultaneously [1]. However, there are trauma mechanisms that have been reported to result in injuries analogous to the presented injury, the most notable being the “clay-shoveler's fracture” [3]. First described in 1940, the “clay-shoveler’s fracture” describes an avulsion fracture of the lower cervical or upper thoracic spinous process, typically C6 or C7. This fracture type received its name due to the high occurrence of this injury in clay shovelers in Australia, who repeatedly threw clay over their shoulders with high levels of momentum [3]. The cause of the injury was believed to be an avulsion fracture caused by sticky clay and forces on the supraspinous ligaments [3]. In later case reports, similar trauma mechanisms that led to related injuries in volleyball players and horse riders were described [4, 5].

We also considered stress fractures resulting from repetitive movements and accumulative stress on bone and ligaments to explain the trauma in the patient in our study [6]. These fractures are mostly observed in runners or soldiers on long marches, are induced by repetitive movements over time, and are considered fatigue-induced fractures. They mostly present as hairline fractures without dislocation [6]. In this case report, we describe a patient who developed a fracture in the seventh cervical vertebra while playing a VR game.

This case report aims to shed light on a new trauma mechanism caused by commonly used VR headsets.

## Case presentation

The Caucasian patient presented to our university clinic with pain and swelling in the lower cervical spine. Before the pain occurred, the patient was playing a VR video game involving combinations of shoulder, arm and head movements to rhythmic visual and musical triggers. He felt sudden pain between his shoulder blades while playing and turning his head rapidly. The patient was not able to point out the exact movement that led to the pain. He reported no falls or collisions with his surroundings. Neurological deficits were not present when he was first admitted to our hospital. Preexisting comorbidities were not present in the 31-year-old male. No history of regular medication use or drug abuse was recorded. The pain level was bearable without painkillers (visual analog scale [VAS] score 3–4). No other injuries or deformities of the spine were detected in the medical records. The only prerecorded injury was a patellar fracture without impact on the momentary resilience or range of motion of the affected joint. No fever or other inflammatory symptoms were present. In anamnesis, the patient claimed to play VR games for 1 to 4 hours on an almost daily basis.

**Fig. 1:**
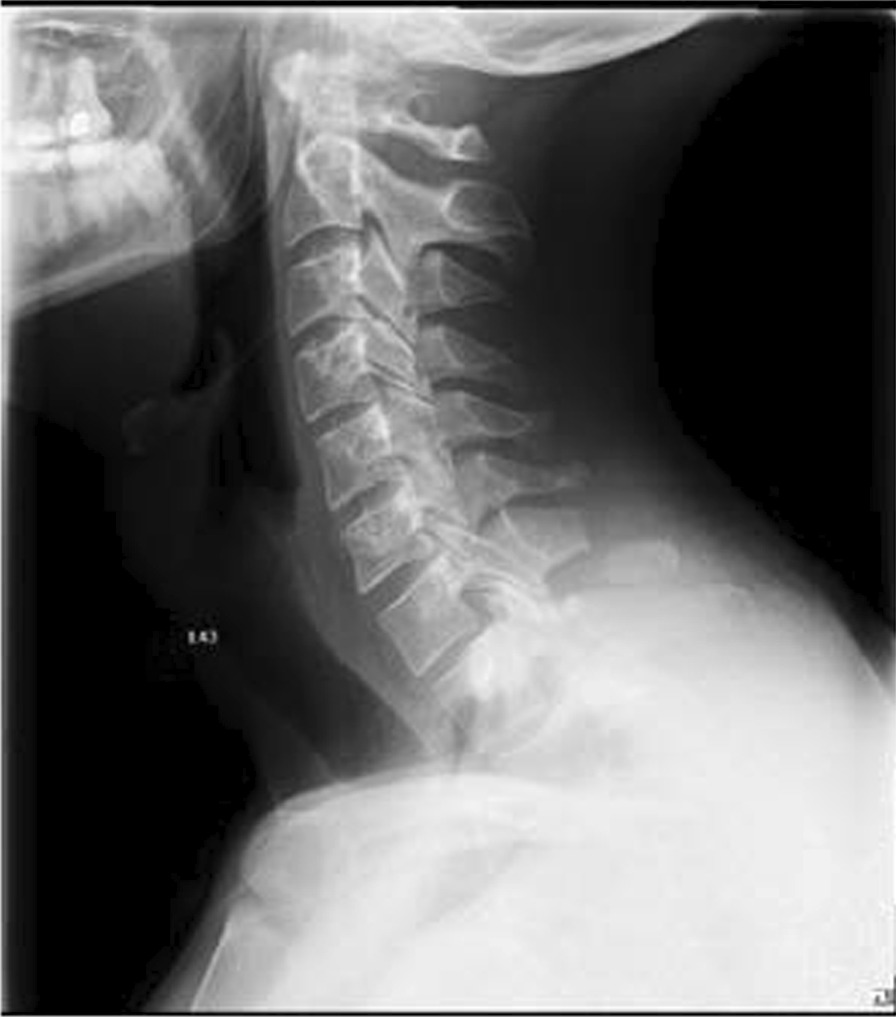
Lateral neck X-ray taken at admission. X-ray, computed tomography and magnetic resonance imaging taken from patient folder with the patient’s consent

**Fig. 2:**
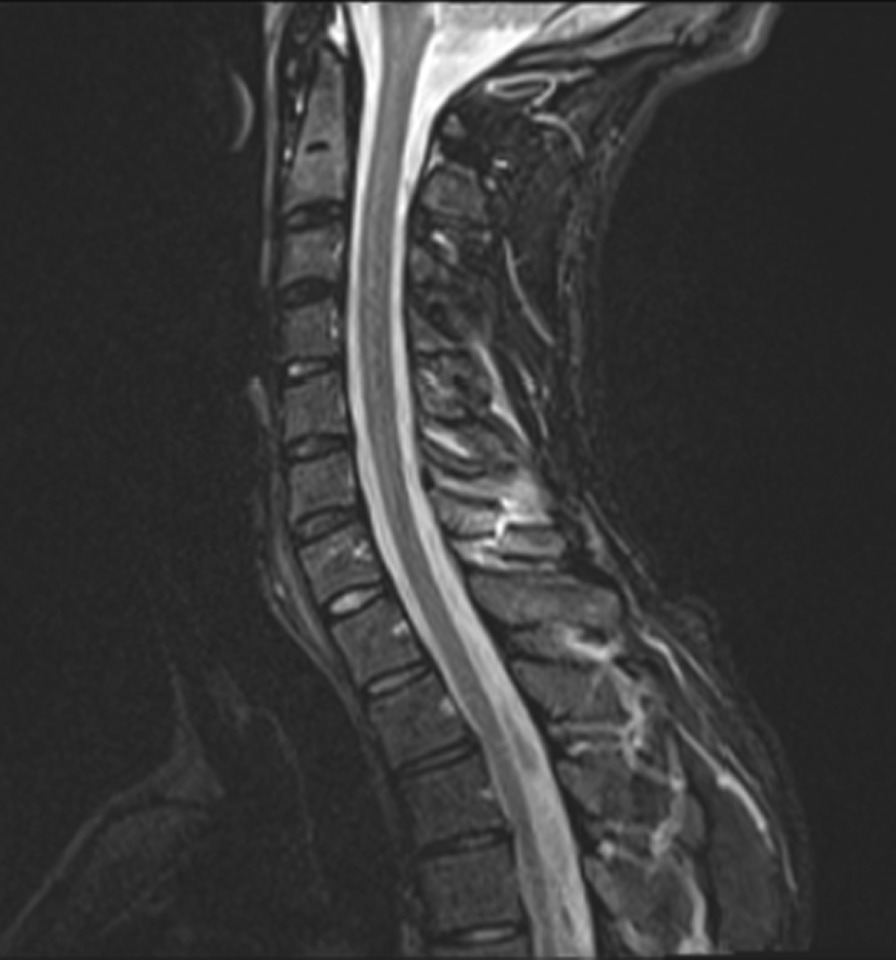
Lateral magnetic resonance imaging (MRI) turbo inversion recovery magnitude (TIRM) sequence. X-ray, computed tomography and MRI taken from patient folder with the patient’s consent

**Fig 3:**
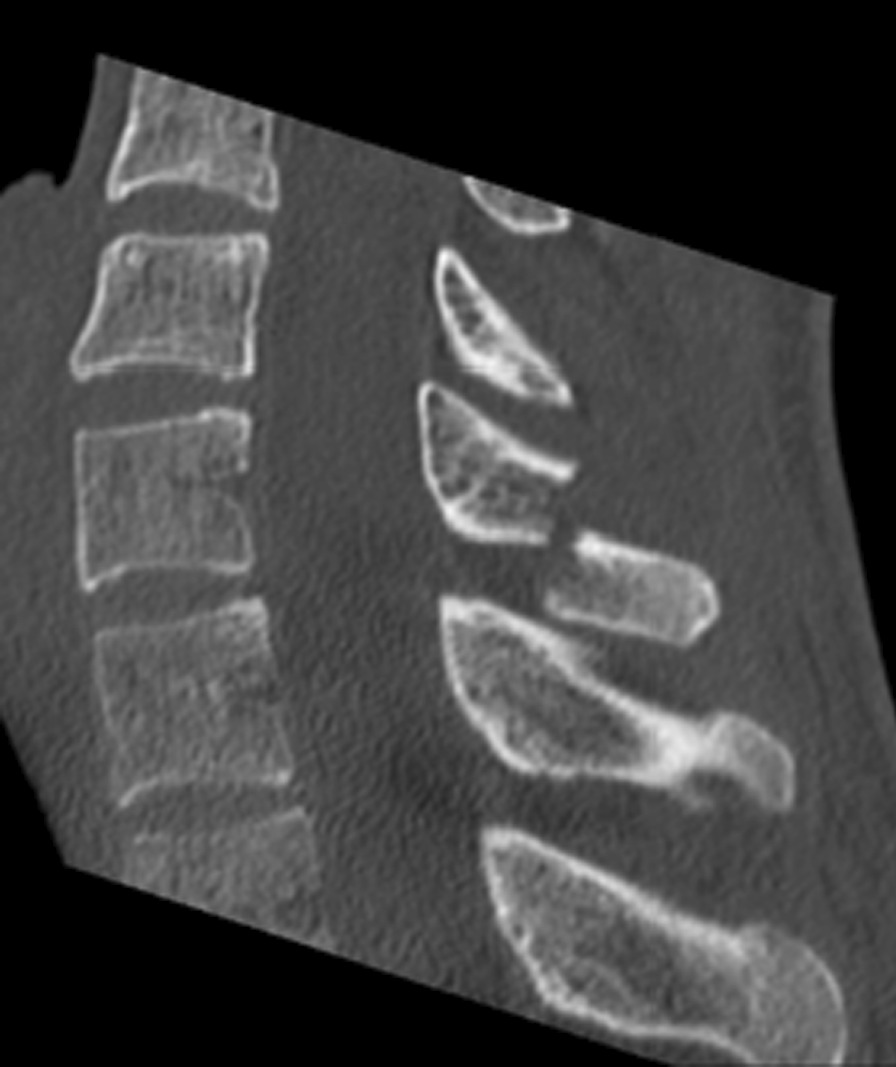
Lateral computed tomography (CT) scan, X-ray, CT and magnetic resonance imaging taken from patient folder with the patient’s consent

After the clinical examination and radiological diagnostics via computed tomography (CT) and conventional X-ray scans of the cervical spine, we found a dislocated traumatic fracture of the spinous process of the seventh cervical vertebra (C7:A0 according to the AO classification system) [7] (Figs. 1, 2). For comprehensive diagnostics, a magnetic resonance imaging (MRI) scan of the cervical spine was carried out. The MRI findings showed neither signs of soft tissue edema around the fracture nor spinal stenosis. Furthermore, no rupture of the anterior or posterior longitudinal ligament was detected (Fig. 3)

Additionally, due to the unusual trauma mechanism, we conducted laboratory investigations to exclude the possibility of preexisting osteoporosis.

The patient showed no signs of osteoporosis in the blood tests, with normal levels of serum 1,25-hydroxyvitamin D (vitamin D), parathyroid hormone (PTH), ß-CrossLaps and alkaline bone phosphatase. The CT scan showed normal bone density in the seventh cervical and surrounding vertebrae, as a mean of 315 Hounsfield units (HU) was measured. To limit radiation exposure, we did not perform a dual-energy X-ray absorptiometry (DEXA) scan [8, 9]. The patient received a conservative therapy regimen with pain therapy and immobilization of the cervical spine in a semirigid collar [10]. During hospitalization, the patient recovered under conservative treatment, and no signs of neurological deficits were present at any time.

After concluding diagnostics, the patient was discharged with outpatient treatment.

After hospitalization, outpatient controls were conducted at 4, 6 and 12 weeks. The patient had a good general condition throughout the recovery process, without additional symptoms. After 4 weeks, the patient no longer needed pain medication.

The radiological controls showed no further dislocation of the fracture, and the patient tapered the use of the semirigid collar at 6 weeks after trauma.

He showed no hypermobility or pain during fast movements of the cervical spine, with full range of motion. In the following 6 weeks, no pain or neurological deficits were reported by the patient. After 12 weeks, the patient was active and was able to return to sports.

## Discussion

Rapid movements during VR gaming can lead to injuries and should not be underrated. In addition to rapid movements, the additional weight of VR headsets (460–610 g) as well as the decoupling of audiovisual stimuli from the actual proprioceptive information should be considered. We considered stress and avulsion fractures as possible fracture types responsible for the injury in our patient.

The weight of the VR headset and the controllers used by both hands are not comparable to the weight of wet clay and the metal and wood shovels used by workers in the past.

Therefore, we think an avulsion is unlikely to be the underlying cause of the injury in the patient in our study. Since the patient had been playing VR games for many hours weekly with lightweight devices in his hands and on his head, we conclude that a stress-type fracture seems to be the more likely reason for the dislocated fracture of the spinous process from the seventh cervical vertebra. This theory is supported by the absence of edema and soft tissue defects in the MRI scan. The repetitive movements and intense gaming habits could have led to a fatigue fracture. This possibility is underlined by the negative HU measurement in the CT scan of the upper spine, as well as by the inconspicuous osteoporosis laboratory results. According to previous studies in the literature, conservative treatment with a semi-rigid collar usually leads to a rapid recovery without permanent limitations to the cervical range of motion or lasting pain in the lower cervical spine.

In 2016-2018, approximately 170 million active users of similar headsets were registered worldwide [11]. In the future, this new type of trauma can have greater relevance due to the increasingly widespread use of this technology among gamers and other users.

## Conclusion

In patients presenting with pain in the cervical spine after VR gaming, a stress fracture should be considered, and adequate diagnostics should be initiated. To our knowledge, this is the first documented case of a VR gaming stress fracture, and more cases need to be documented to clarify the significance of this type of trauma mechanism. We are excited for future developments.

## Data Availability

Imaging data are available. The other data for the endpoints that are available concern the written anamnesis and physical examination.

## Abbreviations

VR: Virtual reality
CT: Computed tomography
MRI: Magnetic resonance imaging
DEXA: Dual-energy X-ray absorptiometry
Vitamin D: Serum 1,25-hydroxyvitamin D
PTH: Parathyroid hormone
VAS: Visual analog scale
HU: Hounsfield unit
TIRM: Turbo inversion recovery magnitude
